# Construction and validation of a predictive model for acute pain after total knee arthroplasty

**DOI:** 10.1097/MD.0000000000040963

**Published:** 2026-02-20

**Authors:** Yi Yang

**Affiliations:** a Ya’an Hospital of Traditional Chinese Medicine, Ya’an, Sichuan Province, China.

**Keywords:** acute, arthroplasty, knee, nomogram, pain

## Abstract

The aim was to investigate the independent risk factors for acute pain after total knee arthroplasty, and to build a nomogram prediction model accordingly. Data were collected from total knee replacement patients in our hospital from June 2022 to December 2023, and independent risk factors for acute pain after total knee replacement were identified using univariate and multivariate logistic regression analyses, and the corresponding nomograms were established. The performance of the model was evaluated by plotting the working characteristic curves of the subjects and calculating the area under the curve, and the model performance was evaluated by using calibration curves and decision curve analyses in order to further enhance the reliability of the validation results. To further improve the reliability of the validation results, internal validation was performed using Bootstrap with 10-fold cross-validation rows, and the clinical utility of the model was assessed using calibration curve and decision curve analysis. A total of 486 total knee replacement patients were enrolled in the study, and 149 patients with acute pain after total knee replacement, with an incidence rate of 30.66%. After univariate and multivariate logistic regression analyses, a total of 5 variables were identified as independent risk factors for acute pain after total knee arthroplasty: body mass index > 24 kg/m^2^ (odds ratio [OR]: 1.930; 95% confidence interval [CI]: 1.032–3.917), diabetes (OR: 3.256; 95% CI: 1.106–7.961), placement of drainage tube (OR: 5.327; 95% CI: 1.236–10.237), operative time >2 hours (OR: 4.378; 95% CI: 1.237–9.372), and moderate-to-severe pain fear (OR: 7.665; 95% CI: 1.155–13.442). The nomogram constructed in this study for acute pain after total knee arthroplasty has good predictive accuracy and helps physicians to intervene in advance for patients at high risk of acute pain after total knee arthroplasty.

## 1. Introduction

Acute postoperative pain, as a series of physiological, psychological, and behavioral responses produced by the organism after surgical stimulation, mainly reaches its peak within 24 to 72 hours after surgery.^[1,2]^ It not only affects the patient’s postoperative recovery process and prolongs hospitalization time, but also is a potential risk factor for the development of chronic postoperative pain.^[3]^ Postoperative pain management is particularly important in total knee arthroplasty, which is a mainstream option for the treatment of advanced osteoarthritis of the knee.^[4]^ Previous studies have noted that approximately 40% of total knee arthroplasty patients experience moderate-to-severe acute pain postoperatively, highlighting the urgency of identifying and intervening on factors that influence this.^[5]^

Currently, studies on acute pain after total knee arthroplasty mostly focus on the exploration of a single risk factor, while there is a relative lack of comprehensive analysis of the interaction of multiple factors affecting the risk of perioperative blood transfusion. In view of this, an in-depth investigation of the multifactorial impact of acute pain after total knee replacement should not only focus on the physiological state of the individual, but also take into account the patient’s lifestyle habits and underlying disease status. By constructing a multifactorial model to analyze the interactions between factors and their combined effects on the risk of acute pain after total knee arthroplasty, it can help to identify the high-risk group more accurately, and provide a scientific basis for the development of individualized prevention and early intervention strategies.

Clinical predictive models provide an estimate of the probability of occurrence of a specific disease or clinical event by analyzing the interrelationships among predictor variables. Nomogram, as a visual presentation of such predictive models, has been successfully applied in several healthcare domains for its simplicity, intuition, and accuracy, for example, prediction of transfusion requirements after fracture surgery^[6]^ and assessment of the formation of deep vein thrombosis in the lower extremities after fracture surgery risk,^[7]^ both of which have shown excellent predictive capabilities.

The focus of this study was to explore the independent risk factors for acute pain after total knee arthroplasty and to construct a nomogram prediction model based on them. By systematically analyzing the multiple variables affecting postoperative pain, we aim to provide a practical tool to help clinicians accurately predict the likelihood of postoperative pain in individual patients, which in turn guides the development of personalized pain management strategies. The establishment of this model is expected to provide strong support for optimizing postoperative pain control after total knee arthroplasty, shortening hospitalization time, and improving the quality of patient recovery.

## 2. Data and methods

### 2.1. Data sources and data collection

In this study, the medical data of patients hospitalized in our hospital who underwent total knee replacement surgery during the period from June 2022 to December 2023 were reviewed retrospectively. The study was retrospective in nature and complied with the relevant standards of the hospital ethics committee.

### 2.2. Inclusion exclusion criteria

The selection of study subjects followed a set of clear criteria to ensure homogeneity of the sample and accuracy of the study. The inclusion criteria included: patients with a diagnosis of osteoarthritis of the knee and only receiving unilateral total knee arthroplasty as a treatment, and patients were required to have complete clinical records. The exclusion criteria, on the other hand, were designed to eliminate factors that might interfere with the results of the study, specifically: patients with a history of knee surgery, the presence of severe cardiovascular disease, a diagnosis of malignancy, and cases where bilateral total knee arthroplasty was planned or had been performed. Through this series of screening criteria, we aimed to construct a purer and more representative patient population, thus improving the reliability and validity of the study results.

### 2.3. Collection of relevant variables

Collecting information about patients with osteoarthritis of the knee requires not only focusing on the individual’s physiologic status, but also considering the patient’s lifestyle habits and underlying disease status, as well as detailed information about the surgery. Therefore, the included information included patients’ age, gender, body mass index (BMI), whether they suffered from hypertension, diabetes mellitus, hyperlipidemia, cardiovascular disease, depression, anxiety, as well as information related to the long-term use of pain medication, preoperative prophylaxis for pain relief, preoperative resting Numeric Rating Scale scores, pain tendency scores, the American Society of Anesthesiologists Physical Status classification, operation time, intraoperative bleeding, placement of drains, and use of analgesic pumps in the postoperative period.

### 2.4. Statistical analysis

To construct and validate the prediction model, we used stratified random sampling method using R software version 4.2.1, and the acquired patient data were assigned to the modeling group and the validation group in the ratio of 70% to 30%, respectively. In the modeling group, we performed a preliminary univariate analysis using SPSS 26.0 software to explore whether there was a significant difference between the different variables, and for categorical data, we used the chi-square test for statistical analysis. Subsequently, variables that showed statistical significance (*P* < .05) in the univariate analysis were further incorporated into a multivariate logistic regression model to identify independent risk factors for acute pain after total knee arthroplasty, a process that also used *P* < .05 as the criterion for significance. Next, after identifying the independent risk factors, we utilized the correlation function in the R software to construct a nomogram prediction chart based on the results of the multivariate logistic regression analysis. This graph not only visualizes the degree of contribution of each risk factor to the prediction results, but also facilitates clinicians to perform rapid prediction calculations. In order to assess the prediction accuracy and stability of the model, we plotted the receiver operating characteristic curves in the modeling and validation groups and calculated the area under the curve, and the closer the value of the area under the curve is to 1, the better the prediction performance of the model. To further improve the reliability of the validation results, Bootstrap (number of samples: 1000) combined with 10-fold cross-validation rows was used for internal validation. In addition, we also plotted calibration curves and decision curves, the former used to test the consistency of the model predicted values with the actual observed values, and the latter helped to assess the clinical utility of the model under different decision thresholds, providing a quantitative basis for clinical decision making. Through this series of statistical analyses and model validation, our goal was to develop an accurate and reliable prediction tool to optimize the management strategy of acute pain after total knee arthroplasty.

## 3. Results

### 3.1. General information

A total of 486 patients who underwent total knee arthroplasty were enrolled in this study, and a total of 149 patients with acute pain after total knee arthroplasty occurred, with an incidence rate of 30.66%. According to the ratio of 7:3, 340 and 146 patients were randomly divided into modeling and validation groups.

### 3.2. Independent risk factors for acute pain after total knee arthroplasty

In the modeling group, 18 variables were analyzed by univariate logistic regression analysis, and the results showed that 10 variables were potential risk factors for acute pain after total knee arthroplasty, including gender, BMI, diabetes mellitus, depression, anxiety, preoperative prophylaxis for pain relief, pain tendency score, duration of the operation, placement of drain, and use of an analgesic pump after the operation (Table 1). Multifactorial logistic regression analysis was performed to determine BMI > 24 kg/m^2^ (odds ratio [OR]: 1.930; 95% confidence interval [CI]: 1.032–3.917), diabetes mellitus (OR: 3.256; 95% CI: 1.106–7.961), placement of drainage tubes (OR: 5.327; 95% CI: 1.236–10.237), operative time >2 hours (OR: 4.378; 95% CI: 1.237–9.372), and moderate-to-severe pain fear (OR: 7.665; 95% CI: 1.155–13.442) were the independent risk factors for acute pain after total knee arthroplasty (Table 2).

**Table 1:** Univariate analysis of acute pain after total knee arthroplasty.

Risk factor	Acute pain group (n = 104)	No acute pain group (n = 236)	*P* value
Age			.114
≤70 yr	74	147	
>70 yr	30	89	
Sex			.007
Male	33	112	
Female	71	124	
BMI			.007
≤24 kg/m^2^	43	135	
>24 kg/m^2^	61	101	
Hypertension			.096
Yes	56	104	
No	48	132	
Diabetes			.019
Yes	52	86	
No	52	150	
Hyperlipidemia			.900
Yes	24	53	
No	80	183	
Cardiovascular disease			.982
Yes	25	57	
No	79	179	
Depression			.035
Yes	29	42	
No	75	194	
Anxiety			.034
Yes	28	40	
No	76	196	
Long-term use of painkillers			.635
Yes	86	190	
No	18	46	
Preoperative preventive pain relief			.025
Yes	67	121	
No	37	115	
Preoperative resting NRS score			.642
≤4 points	46	98	
>4 points	58	138	
Pain tendency score			.006
No or mild pain fear	60	172	
Moderate-to-severe pain fear	44	64	
ASA classification			.285
≤2	68	168	
>2	36	68	
Surgical time			.033
≤2 h	64	171	
>2 h	40	63	
Intraoperative bleeding			.460
≤50 mL	49	101	
>50 mL	55	135	
Placement of drainage tube			.001
Yes	38	46	
No	66	190	
Postoperative use of analgesic pumps			.026
Yes	82	208	
No	22	28	

ASA = American Society of Anesthesiologists Physical Status, BMI = body mass index, NRS = Numeric Rating Scale.

**Table 2:** Multivariate analysis of acute pain after total knee arthroplasty.

Risk factors	β	Wald χ^2^	*P*value	OR	95% CI
BMI > 24 kg/m^2^	1.348	7.092	.045	1.930	1.032–3.917
Diabetes	0.372	9.102	.024	3.256	1.106–7.961
Placement of drainage tube	0.142	10.952	.006	5.327	1.236–10.237
Surgical time >2 h	1.375	12.573	.001	4.378	1.237–9.372
Fear of moderate-to-severe pain	0.216	11.542	.037	7.665	1.155–13.442

BMI = body mass index, CI = confidence interval, OR = odds ratio.

### 3.3. Nomogram development and validation

**Figure 1.**
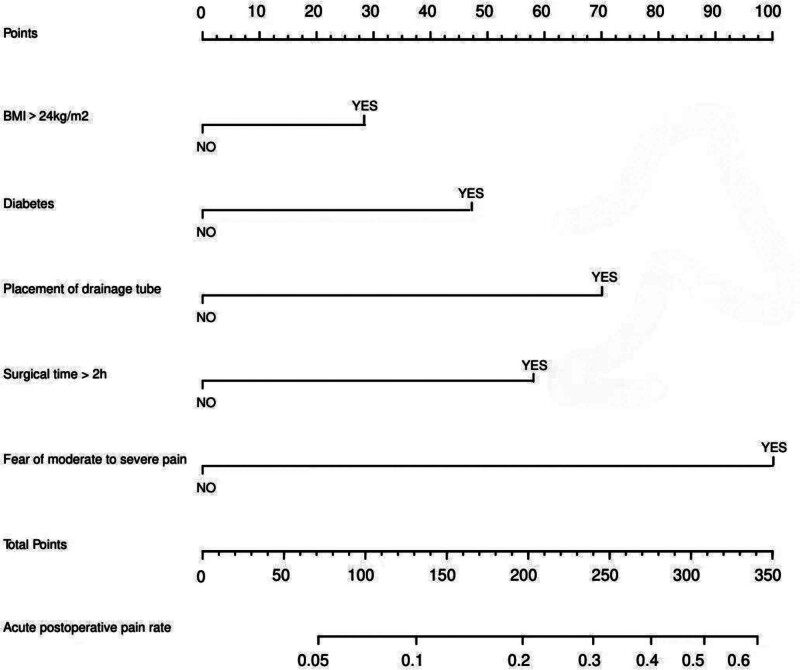
Nomogram prediction model for acute pain after total knee arthroplasty.

**Figure 2.**
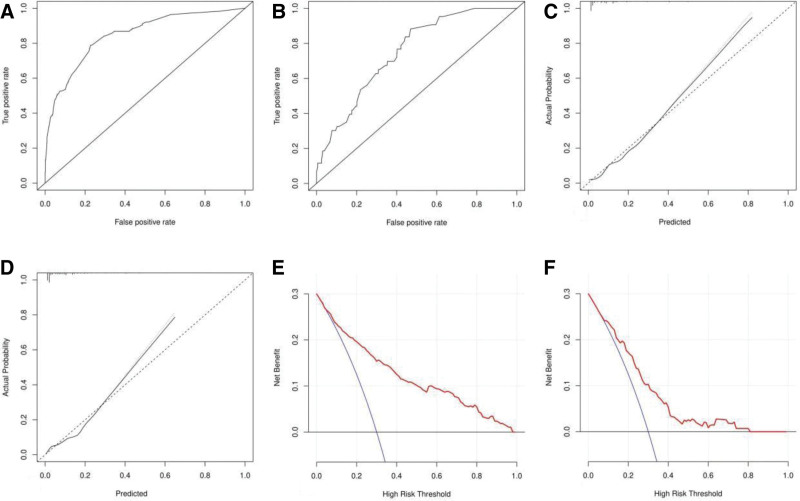
Receiver operating characteristic curves of nomograms for predicting acute pain after total knee replacement in the modeling group (A) and validation group (B). Nomogram calibration curves for predicting acute pain after total knee replacement in the modeling set (C) and validation set (D). Decision curves for nomograms used to predict acute pain after total knee replacement in modeling group (E) and validation group (F).

A nomogram was developed using the screened independent risk factors and used to predict the risk of acute pain after total knee arthroplasty (Fig. 1). Receiver operating characteristic curves were then plotted for the modeling and validation groups, and the corresponding areas under the curves were calculated to be 0.846 and 0.750 (Fig. 2A and B). The results of the Hosmer–Lemeshow goodness-of-fit test show that χ^2^ = 7.304, *P* = .489 for the modeling group and χ^2^ = 7.256, *P* = .496 for the validation group, which indicates that the models developed are highly consistent. In order to further improve the reliability of the validation results, internal validation was performed using Bootstrap combined with 10-fold cross-validation rows, and the results showed that the area under the curve of the receiver operating characteristic curve was 0.831, the sensitivity was 78.5%, and the specificity was 79.6%, which indicated that the model performed well in terms of fitting. In addition, the calibration curves are plotted, indicating that the nomogram predicts the risk in good agreement with the actual occurrence of the risk, and has a good predictive ability (Fig. 2C and D). Also, the decision curve showed that nomogram had good predictive ability (Fig. 2E and F).

## 4. Discussion

Acute postoperative pain is a common complication after knee arthroplasty, affecting patients’ postoperative recovery and prognosis.^[5,8,9]^ The results of this study showed that the incidence of acute postoperative pain after knee arthroplasty was 30.66%, which is similar to the findings of Pace et al.^[10]^ The incidence of acute postoperative pain is closely related to demographic sociological factors, psychological factors, and surgical and anesthesia-related factors.^[11,12]^ Several previous studies have explored the risk factors for acute postoperative pain after total knee arthroplasty, but they have analyzed a single factor.^[13–15]^ In the current study, we used predictors that are common and easily identified in clinical practice and developed this nomogram prediction model based on the 5 independent risk factors identified, and after model validation it was determined that the model developed in this study had good predictive ability.

In this study, BMI > 24 kg/m^2^ was found to be a risk factor for acute pain after total knee arthroplasty. Obese patients are usually accompanied by a high BMI, which not only increases the burden on the joints, but also leads to an abnormal distribution of pressure on the muscles and soft tissues around the knee joint, which exacerbates postoperative pain.^[16]^ It was found that chronic low-grade inflammatory response in obese state may further aggravate postoperative inflammation, and excessive release of inflammatory mediators such as cytokines and chemokines not only promotes the transmission of pain signals, but also may prolong the duration of inflammatory response, leading to the persistence of pain.^[17]^ In addition, high levels of adipocytokines, such as leptin and lipocalin, are present in the adipose tissue of obese patients, and an imbalance of these substances may affect the regulatory mechanisms of pain perception, thereby increasing sensitivity to pain.^[18]^ It has been demonstrated that obesity may also indirectly influence the postoperative pain management and recovery process through metabolic syndrome-related cardiovascular and endocrine alterations.^[19]^ Based on these findings, more emphasis should be placed on risk assessment and individualized treatment strategies for obese patients, including early weight management and pain intervention, in the future management of total knee arthroplasty preoperatively and postoperatively, with the aim of improving surgical outcomes and patient quality of life.

In this study, diabetes mellitus was found to be a risk factor for acute pain after total knee arthroplasty. Diabetic patients can suffer from peripheral neuropathy due to a chronic hyperglycemic state, manifested by slowed nerve conduction and nerve dysfunction, which directly exacerbates the sensitivity of postoperative pain perception.^[20]^ In addition, diabetes-related microangiopathy and ischemic state may affect blood perfusion to the surgical area, delaying tissue healing, which in turn prolongs the duration of pain.^[21]^ It has been found that a hyperglycemic environment promotes an inflammatory response, and overexpression of inflammatory cytokines such as tumor necrosis factor alpha and interleukin-1 beta not only exacerbates pain, but may also enhance pain signaling through activation of the pain transduction pathway, creating a vicious cycle.^[22]^ It has been demonstrated that diabetes is also associated with adipose tissue dysfunction, and the abnormal regulation of adipocytokines may indirectly affect the pain threshold and the complexity of pain management.^[23]^ Based on these findings, in future clinical practice, glycemic control and pain management should be strengthened for diabetic patients undergoing total knee arthroplasty, including early intervention and individualized treatment strategies to optimize surgical outcomes and promote patient recovery. Meanwhile, comprehensive preoperative evaluation and close postoperative monitoring are essential for the prevention and management of acute postoperative pain, which can help improve the overall surgical experience and prognosis of diabetic patients.

In this study, placement of drains was found to be a risk factor for acute pain after total knee arthroplasty. It has been found that the placement of drains produces mechanical irritation to the surrounding tissues, especially during joint movement or positional changes, which may lead to the activation of pain receptors that can cause or exacerbate postoperative pain.^[24]^ In addition, the presence of drains may prolong the healing time of surgical wounds, increase the persistence of local inflammatory responses, and the excessive release of inflammatory mediators further exacerbates pain sensations. It has been demonstrated that drains may be a potential route for bacterial infection, and even under strict asepsis, minor bacterial contamination may still trigger a local or systemic inflammatory response, which in turn affects pain management.^[25]^ Therefore, this study highlights the need to weigh the necessity of drains in total knee arthroplasty, especially in patients at low risk of bleeding, and reducing or avoiding the unnecessary use of drains may help to reduce acute postoperative pain. Meanwhile, for patients who require drains, optimizing the placement and timing of drains, using gentler materials, and enhancing postoperative pain management and patient psychological support are all key measures to improve the postoperative experience and promote recovery. By comprehensively considering surgical technique, pain management, and patient education, pain control strategies after total knee arthroplasty can be further optimized to improve patient satisfaction and treatment outcomes.

In this study, prolonged surgical time was found to be a risk factor for acute pain after total knee arthroplasty. It was found that prolonged surgical time directly increased the degree of tissue damage, leading to increased physical stimulation of the surgical wound and activation of more injury receptors, which triggered or exacerbated postoperative pain.^[26]^ In addition, tissue ischemia-reperfusion injury is more pronounced during prolonged surgical procedures, which promotes the release of inflammatory mediators such as cytokines, prostaglandins, and leukotrienes, which play a key role in the local inflammatory response and not only directly stimulate pain receptors, but also enhance pain signaling and prolong the duration of pain by lowering the pain threshold.^[27]^ Some studies have demonstrated that prolonged surgery may also cause an increase in the body’s stress response, such as elevated cortisol levels, which not only affects pain perception, but may also suppress immune function and increase the risk of infection, further complicating pain management.^[26]^ Therefore, surgeons should optimize the surgical process and improve surgical efficiency in order to reduce unnecessary surgical time. Meanwhile, more aggressive pain prevention measures, including the early application of multimodal analgesic strategies, should be taken to reduce acute postoperative pain in patients who are expected to undergo prolonged surgery.

In this study, moderate-to-severe pain fear was found to be a risk factor for acute pain after total knee arthroplasty. It has been found that pain fear not only affects the patient’s emotional state at the psychological level, but also acts directly on pain perception and processing through psycho-physiological mechanisms.^[28]^ Studies have demonstrated that intense fear of pain can activate emotional centers in the brain, such as the amygdala and the anterior cingulate gyrus, which are closely related to the modulation of pain perception, and that overactivation may lead to a decrease in the pain threshold and make patients more sensitive to pain.^[29]^ In addition, fear of pain may also trigger a stress response that increases the release of cortisol and catecholamines, further exacerbating pain perception.^[30]^ In addition, this psychological stress may influence the immune and inflammatory response and promote the release of inflammatory mediators, thus contributing to postoperative inflammation and pain.^[31]^ Therefore, the importance of preoperative psychological preparation and pain education is emphasized. Preoperative psychological interventions, such as cognitive behavioral therapy and relaxation training, can effectively reduce patients’ fear of pain and increase their anticipation and confidence in postoperative pain management. Meanwhile, multidisciplinary teamwork, including close collaboration among anesthesiology, orthopedics, and psychology, is essential to provide comprehensive pain management and patient support. In postoperative pain management, more attention should be paid to patients’ psychological status, timely recognition and management of pain fear, and individualized analgesic strategies, including a combination of pharmacological and nonpharmacological treatments, with the aim of achieving optimal pain control. By comprehensively considering patients’ psychological needs and optimizing the pain management process, the patient experience and recovery process after total knee arthroplasty can be significantly improved, promoting better clinical outcomes.

In clinical applications, the process of using the nomogram covers the collection of patient data, the input of data to calculate individualized risk scores, and the development of personalized management strategies based on the scores. However, this process faces a number of challenges, such as the need to train medical staff to ensure that they can properly understand and use the nomogram, as well as the fact that the quality and completeness of the data are critical to the accuracy of the nomogram, and that its applicability in different healthcare environments needs to be further validated. In addition, improving the clinical acceptance of the nomogram, dealing with possible resistance, and regularly updating and maintaining the tool are essential for the effective application of the nomogram.

This study successfully constructed and validated a prediction model for acute pain after total knee arthroplasty, an achievement that fills the gap of population-specific risk assessment tools in related fields. The results show that the model can effectively identify high-risk patients with specific clinical characteristics, providing clinicians with an accurate risk assessment tool to optimize preventive measures and personalized management strategies.

The innovative nature of this study is reflected in the use of large sample data and advanced statistical methods, which not only improved the reliability and applicability of the model, but also provided new perspectives on the early identification of acute pain after total knee arthroplasty, and emphasized the need for further research in multicenter validation and model optimization.

Directions for future research should focus on validating the applicability of the model in different populations and healthcare settings and exploring other potential risk factors. Based on the results of this study, further development of interventions to reduce the incidence of acute pain after total knee arthroplasty is important for improving the quality of clinical care.

Nevertheless, this study has some limitations. First, due to the retrospective design, there may be missing data and accuracy issues. Second, the study was conducted in a tertiary care hospital with more complex patients and possible selection bias. Finally, as a risk prediction model developed in a single center, its validity needs to be further validated by large-scale, multicenter studies.

## 5. Conclusion

The results of this study indicate that BMI > 24 kg/m^2^, diabetes, placement of drain, operation time >2 hours, and moderate-to-severe pain fear are risk factors for acute pain after total knee arthroplasty. The nomogram of acute pain after total knee arthroplasty constructed in this study has good predictive accuracy and helps physicians to intervene in advance in patients at high risk of acute pain after total knee arthroplasty.

## Acknowledgments

I would like to thank Deng Guanghua for his guidance in analyzing the data and submitting my thesis.

## Author contributions

**Conceptualization:** Yi Yang.

**Data curation:** Yi Yang.

**Formal analysis:** Yi Yang.

**Funding acquisition:** Yi Yang.

**Investigation:** Yi Yang.

**Methodology:** Yi Yang.

**Project administration:** Yi Yang.

**Resources:** Yi Yang.

**Software:** Yi Yang.

**Supervision:** Yi Yang.

**Validation:** Yi Yang.

**Visualization:** Yi Yang.

**Writing – original draft:** Yi Yang.

**Writing – review & editing:** Yi Yang.
